# The Impact of AI Usage on Innovation Behavior at Work: The Moderating Role of Openness and Job Complexity

**DOI:** 10.3390/bs15040491

**Published:** 2025-04-08

**Authors:** Qichao Zhang, Ganli Liao, Xueying Ran, Feiwen Wang

**Affiliations:** Business School, Beijing Information Science and Technology University, Beijing 102206, China; zhangqichao@bistu.edu.cn (Q.Z.); xueying_ran@bistu.edu.cn (X.R.); bistu_wfw@163.com (F.W.)

**Keywords:** AI usage, self-efficacy, innovation behavior, openness, job complexity

## Abstract

In the context of the digital transformation era, the extensive application of artificial intelligence (AI) is profoundly altering the workplace environment, thereby underscoring the critical need to elucidate its impact on employee innovation behavior. Such insights are essential for optimizing human resource management and enhancing organizational competitiveness. Grounded in cognitive evaluation theory, this study explores the underlying mechanisms through which AI usage influences employee innovation behavior and develops an integrated theoretical model that incorporates both employee personality traits and job characteristics. A two-wave questionnaire survey was conducted, and hierarchical regression analysis was employed to test the hypotheses using a sample of 339 employees from 13 manufacturing enterprises in China. The findings reveal that AI usage is positively associated with employee innovation behavior, with self-efficacy serving as a significant mediator. Furthermore, openness and job complexity positively moderate the relationship between AI usage and self-efficacy, thereby facilitating innovative behavior. Additionally, a moderated mediation mechanism was identified. The conclusions of this study not only enrich the theoretical understanding of how AI impacts employee innovation behavior but also offer practical guidance for organizations on leveraging AI to foster innovation during digital transformation.

## 1. Introduction

Innovation constitutes a critical determinant in establishing and sustaining competitive advantages, with contemporary enterprises increasingly relying on innovative capabilities to navigate dynamic business landscape (Roberts & Candi, 2024). To sustain competitive advantage in volatile markets, organizations must prioritize continuous innovation, with employee innovative behavior serving as a critical driver of organizational innovation outcomes (Ahn et al., 2025; Xie & Tian, 2025; Gao & Gao, 2024). Employees demonstrating innovative behaviors excel in identifying process optimization opportunities, whether through creative workflow enhancements, novel service delivery methods, or personalized customer experience innovations. These behaviors exhibit multifaceted value propositions, driving digital transformation, advancing green sustainability initiatives, fulfilling corporate social responsibilities, and enhancing organizational resilience during crises (Fey & Kock, 2022; Verhoef et al., 2021; Xue et al., 2022). Consequently, investigating the antecedents of employee innovation behavior holds strategic significance for organizations seeking market adaptability, competitive differentiation, service excellence, and operational efficiency gains.

Artificial intelligence, defined as autonomous systems capable of machine learning, reasoning, problem-solving, and decision-making, has emerged as a transformative force across organizational domains including strategic management, operational decision-making, manufacturing processes, and R&D ecosystems (Mariani & Dwivedi, 2024). The rapid evolution of AI technologies not only expands organizational technological capabilities but also necessitates innovative adaptation within reconfigured business ecosystems (Mariani & Dwivedi, 2024). This transformation is particularly pronounced in technology-intensive manufacturing sectors, where AI implementation has fundamentally altered production paradigms, reengineered innovation value chains, and catalyzed business model transformations, concurrently reshaping workforce attitudes and work patterns (N. Jia et al., 2024). Given this paradigm shift, understanding employees’ cognitive appraisals of AI technologies and their subsequent implications for innovation processes becomes imperative for both theoretical advancement and managerial practice.

Emerging scholarship presents divergent perspectives regarding AI’s impact on employee innovation behavior. Proponents highlight AI’s capacity to augment innovation through enhanced information processing, workflow optimization, and personalized competency development (Carter & Wynne, 2024). Empirical evidence further suggests that human–AI collaboration liberates employees from routine tasks, thereby reallocating cognitive resources toward creative endeavors (N. Jia et al., 2024). However, emerging evidence suggests potential innovation inhibition effects (Brougham & Haar, 2018). Their conceptualization of “AI awareness”, defined as employees’ perceived susceptibility to technological displacement, has catalyzed investigations into job insecurity, skill obsolescence anxiety, and over-reliance dynamics, all potentially undermining innovative capacities (Kong et al., 2021). This theoretical discord stems from two critical lacunae: First, conceptual fragmentation plagues existing constructs (e.g., AI awareness vs. STARA awareness), with inconsistent operational definitions and inherent positivity/negativity biases (Ding, 2021). Second, insufficient attention has been paid to boundary conditions, particularly individual differences (e.g., personality traits) and contextual factors (e.g., job characteristics), that may moderate AI–innovation relationships (Z. Li et al., 2024). To address these limitations, this study examined AI usage—defined as the extent to which employees leverage AI technologies to achieve work objectives—as a neutral and objectively manipulated variable.

Drawing on cognitive evaluation theory (CET), we proposed that employees’ cognitive and behavioral outcomes related to AI usage are influenced by boundary conditions, such as personality traits (e.g., openness to experience) and job design factors (e.g., job complexity). Previous research demonstrated that individuals with high openness to experience exhibit greater adaptability to technology and are more likely to embrace AI-driven innovations in workflow (Ryan, 1982). Additionally, the Job Characteristics Model suggests that job complexity—represented by factors such as task variety, significance, and autonomy—moderates employees’ evaluations of AI (Huo et al., 2025). In high-complexity jobs, AI’s empowering potential may be enhanced through automated task support and data-driven decision augmentation. Conversely, low-complexity jobs may exacerbate the risks of job substitution and skills degradation (Y. Li et al., 2024).

This study advances the existing literature by proposing a theoretically integrative framework that examines how AI usage shapes innovation behavior through self-efficacy mechanisms, moderated by openness and job complexity. Focusing on manufacturing professionals—a demographic at the forefront of AI-driven workplace transformation—our research pursued threefold objectives: (1) to illuminate the mediating role of self-efficacy in converting AI utilization into innovation outcomes; (2) to define boundary conditions through which individual traits (openness) and contextual dynamics (job complexity) modulate these relationships; and (3) to deliver actionable strategies for optimizing AI deployment and fostering innovation capabilities in manufacturing sectors. This study makes three pivotal theoretical contributions. First, it crystallizes the intermediary function of self-efficacy in bridging AI usage and innovative behavior. Second, it extends cognitive evaluation theory by revealing how personal dispositions and job characteristics collectively shape AI-induced behavioral outcomes, refining the theoretical understanding of technology–behavior interplay. Finally, it resolves inconsistencies in prior research by proposing a unified framework that underscores the conditional nature of AI’s impact, governed by the synergy between personality traits and occupational complexity.

The remainder of this paper is structured as follows. Section 2 reviews the theoretical foundations, formulates the research hypotheses, and presents the conceptual model. Section 3 details the methodology, including data collected from 339 employees in manufacturing enterprises through a two-stage survey and the measurement of core variables. Section 4 presents the empirical results, using hierarchical regression and bootstrapping methods to test the hypothesized relationships. Section 5 discusses the theoretical and practical implications, addresses limitations, and outlines directions for future research.

## 2. Theoretical Background, Research Hypothesis, and Conceptual Model

### 2.1. Cognitive Evaluation Theory

Cognitive evaluation theory (CET), a sub-theory of self-determination theory (SDT), provides a robust framework for understanding how external technological interventions influence intrinsic motivation and subsequent behavioral outcomes (Hsu, 2022; Ryan, 1982). CET posits that external factors (such as AI systems) enhance intrinsic motivation by fulfilling three innate psychological needs: autonomy (perceived control over tasks), competence (confidence in task mastery), and relatedness (social connectedness) (Hsu, 2022). Notably, the need for competence in CET aligns closely with the concept of self-efficacy in Bandura’s social cognitive theory, as both emphasize individuals’ perceived capability to perform tasks successfully.

In the context of AI-driven transformation, CET has gained increasing relevance in explaining how intelligent technologies affect employees’ motivational states and behavioral responses (Y. Huang & Gursoy, 2024; Lin et al., 2024; Kong et al., 2023). For example, Kong et al. (2023) showed that AI-supported autonomy promotes innovation via enhanced exploratory behavior in the hospitality sector. Building on this foundation, the present study leveraged CET to examine how AI usage enhances employees’ innovation behavior by strengthening perceived competence and autonomy, which in turn reinforce self-efficacy. Furthermore, we extended this framework by introducing individual (openness) and contextual (job complexity) moderators to explore when and for whom AI’s motivational impact is most pronounced.

### 2.2. AI Usage and Innovation Behavior

Artificial intelligence usage (AI usage) has emerged as a prominent research domain in organizational behavior, information management, and innovation studies (Zang et al., 2024). Defined as “the extent to which employees or organizations integrate AI technologies into task execution within work environments”, AI usage transcends mere technological adoption to encompass human–AI collaborative interactions, technology acceptance dynamics, and workflow transformation (Y. Liu et al., 2024). Its operational characteristics are threefold: (1) intelligence, emulating human cognitive functions (e.g., learning, reasoning, and decision-making); (2) adaptivity, dynamically adjusting behaviors to environmental shifts and user demands; and (3) data-driven optimization, leveraging large-scale datasets for continuous system refinement. Contemporary scholarship primarily examines AI usage’s organizational implications, particularly its role in redesigning organizational architectures, enhancing dynamic capabilities, and reshaping employee behaviors (X. Liu & Li, 2025). Recent empirical studies have further explored how AI usage affects employee innovation. For instance, Ahn et al. (2025) found that AI service quality perceptions enhance service innovation in retail contexts. Dong et al. (2025) revealed that AI adoption can both foster and hinder innovative behavior through contrasting stress appraisals. Similarly, Zheng et al. (2025) demonstrated that AI usage promotes innovation via increased autonomy but may also elevate job stress, with motivational orientations moderating these effects. Together, these findings highlight the complex and context-dependent nature of AI’s influence on employee innovation.

Drawing on CET, this study proposed that AI usage may foster innovation behavior through two complementary mechanisms: (1) amplifying competence and autonomy, and (2) restructuring task architectures and feedback systems. First, AI usage elevates perceived competence by providing employees with data-driven decision support, automation of routine tasks, and optimized workflows (Zang et al., 2024) For instance, AI tools enable the rapid analysis of complex datasets, identification of latent problems, and generation of innovative solutions, capabilities that directly strengthen employees’ confidence in task execution (Y. Liu et al., 2024). Concurrently, AI enhances perceived autonomy through intelligent task delegation. In creative domains, AI-generated preliminary designs serve as cognitive scaffolds, allowing employees to focus on high-level ideation and iterative refinement, a synergy that balances efficiency with creativity (N. Jia et al., 2024). Empirical studies confirm that heightened competence and autonomy significantly amplify intrinsic motivation, thereby catalyzing innovation behavior (Puranam et al., 2006).

In parallel, CET further underscores the role of task design and feedback immediacy in sustaining intrinsic motivation (Ryan & Deci, 2000). AI-driven task reconfiguration shifts human labor from repetitive activities (e.g., manufacturing assembly) to complex problem-solving, thereby reducing monotony and expanding opportunities for creative engagement (Liang et al., 2022). Moreover, AI’s real-time feedback mechanisms enable rapid iteration of ideas and strategies (Jarrahi et al., 2023). For example, in R&D settings, AI-powered predictive analytics provide instantaneous performance evaluations, allowing employees to adjust experimental parameters dynamically and optimize innovation outcomes (X. Liu & Li, 2025). Meta-analytic evidence indicates that such immediacy reinforces competence perceptions and accelerates learning cycles, both critical antecedents of innovation (Ogink & Dong, 2019). Accordingly, this study proposed the following research hypotheses:

**H1.**  *AI usage positively influences innovation behavior.*

### 2.3. The Mediating Role of Self-Efficacy

Self-efficacy, defined as an individual’s conviction in their capacity to organize and execute courses of action required to attain designated performance outcomes, constitutes a pivotal psychological mechanism in innovation research (Chen et al., 2025; Gist & Mitchell, 1992). This construct encompasses contextualized confidence in mobilizing existing skills to overcome challenges and achieve creative goals, addressing both confidence in achievement of results and belief in the control of the process. In organizational innovation contexts, self-efficacy manifests as employees’ belief in their ability to generate novel solutions, navigate ambiguous problem spaces, and persist through iterative refinement processes (Eissa & Lester, 2025; G. Jia et al., 2025).

Empirical evidence substantiates self-efficacy’s mediating role between technological exposures and innovative behaviors. Tierney and Farmer’s (2011) seminal work demonstrated its mediation between managerial technology focus and team innovation outcomes, while Choi et al. (2021) established its bridging function in translating leader–member exchange into employee innovation performance. Additionally, longitudinal findings by Francis Xavier and Korunka (2025) suggested that AI-enhanced task environments can elevate creative self-efficacy over time. Complementing this, Zheng et al. (2025) demonstrated that the autonomy afforded by AI systems can reinforce employees’ efficacy beliefs, though such benefits may be attenuated under heightened job stress. Yin et al. (2024) extended this perspective by showing that employees exposed to highly intelligent AI assistants report greater creative self-efficacy, but only when the broader organizational infrastructure is prepared to support AI integration. Collectively, these studies suggest that self-efficacy may play a mediating role between AI and employee innovative behavior.

To better interpret these findings, it is essential to integrate two complementary theoretical perspectives: CET and self-efficacy theory. While CET focuses on how environmental conditions satisfy psychological needs to foster intrinsic motivation, self-efficacy theory centers on individuals’ beliefs in their own ability to execute specific behaviors (Ulfert-Blank & Schmidt, 2022; Xiao & Hew, 2024). Viewed together, these two perspectives offer a complementary lens to understand how AI usage shapes internal psychological processes that translate into innovation-related outcomes.

Drawing on CET, we suggested that external stimuli such as AI usage can strengthen self-efficacy by supporting employees’ needs for autonomy and competence. We argued that AI usage facilitates this process through the following mechanisms. AI technologies operationalize this through three synergistic pathways: (1) AI’s data-driven decision support and automation of routine tasks enable employees to confront complex problems with heightened preparedness, directly elevating task-specific self-efficacy. Grote et al.’s (2024) field experiments in tech firms revealed that real-time AI feedback significantly strengthened engineers’ confidence in tackling innovation bottlenecks. (2) By assuming repetitive subtasks, AI liberates cognitive resources for exploratory innovation activities, thereby enhancing agentic self-efficacy. Tekic and Füller (2023) documented this phenomenon in creative industries, where AI-generated design frameworks empowered designers to experiment with bolder aesthetic choices. (3) CET emphasizes that ongoing competence verification sustains motivation. AI’s adaptive learning systems provide incremental challenges calibrated to users’ skill levels, creating scaffolded mastery experiences that progressively build self-efficacy. Schunk’s (1995) seminal work identified such enactive attainments as the most potent source of efficacy beliefs. Therefore, we formulated the following hypothesis:

**H2.**  *AI usage positively influences self-efficacy.*

While the preceding discussion outlined how AI usage enhances self-efficacy, this psychological shift alone does not fully account for its behavioral implications. Self-efficacy functions not merely as an outcome but as a motivational mechanism that bridges technological environments and employee action (ud din Khan et al., 2023).

This theoretical account is supported by emerging empirical findings. For instance, Yuan et al. (2025) found that structured technological support in professional learning settings enhanced teachers’ self-efficacy, which subsequently improved their classroom innovation practices. Similarly, S. Li et al. (2025) demonstrated that graduate students’ research self-efficacy mediated the relationship between supervisor support and research creativity, reinforcing the pivotal role of efficacy beliefs in academic innovation. K. L. Huang et al. (2024) extended this logic into the AI-enhanced design education context, showing that AIGC integration elevated students’ self-efficacy, which in turn facilitated ideation fluency and novelty. Further, D. Wang et al. (2025) empirically validated creative self-efficacy as a mediating bridge between workplace benign envy and employee innovation, highlighting its centrality across diverse psychological antecedents and outcome domains.

To sum up, these findings suggest that AI usage may not only directly influence innovation behavior but also exert indirect effects through enhanced self-efficacy. In other words, the psychological empowerment afforded by AI may serve as a catalyst that translates technological affordances into creative action. This mediation mechanism aligns with Gist and Mitchell’s (1992) self-efficacy-performance theory, which posits that domain-specific efficacy beliefs mediate between cognitive appraisals of situational factors (e.g., AI usage) and corresponding behavioral outputs (e.g., innovation behavior). Based on the results of the above research and theoretical analysis, this paper proposes the following hypotheses:

**H3.**  *Self-efficacy mediates the positive relationship between AI usage and innovation behavior.*

### 2.4. The Moderating Role of Openness

Openness to experience, a core dimension of the Five-Factor Model of personality, captures individual differences in novelty seeking, aesthetic appreciation, imaginative capacity, and cognitive exploration tendencies (Robinson, 2020; Samimi Dehkordi et al., 2025). Individuals scoring high on this trait exhibit heightened curiosity, divergent thinking patterns, and receptivity to unconventional problem-solving approaches, consistently demonstrating proactive information-seeking behaviors in technological contexts (Wojcieszak et al., 2021). Empirical evidence positions openness as the most robust personality predictor of technology adoption and innovation behavior (Pillai et al., 2024; Ye et al., 2024), with meta-analytic findings indicating its unique explanatory power in AI acceptance models (Q. Wang et al., 2025).

Recent scholarship elucidated openness’s pivotal moderating role in AI-driven innovation processes. Shao et al. (2024) demonstrated that high-openness individuals exhibit greater AI tool adoption rates, primarily driven by their intrinsic motivation to pursue cognitive expansion opportunities afforded by emergent technologies. Complementing this, X. Zhang et al. (2024) revealed through a multilevel analysis of Chinese R&D teams that openness amplifies AI’s innovation benefits via enhanced knowledge recombination capabilities, the cognitive ability to synthesize AI-generated insights with domain expertise. These findings collectively established openness as a critical boundary condition in AI–innovation relationships.

CET provides a theoretical scaffold for understanding this moderation effect. CET posits that personality traits shape primary appraisals of technological stimuli through three interrelated mechanisms: (1) Challenge-Threat Appraisal Asymmetry. High-openness individuals interpret AI usage as cognitive challenges rather than existential threats, focusing on its empowerment potential rather than substitution risks. This positive appraisal framework activates intrinsic motivation for exploratory AI engagement. (2) Cognitive Reconfiguration Flexibility. Openness facilitates adaptive information processing strategies; high scorers treat AI outputs as creative catalysts rather than prescriptive solutions, engaging in the conceptual blending of machine-generated data with tacit knowledge to fuel innovation (H. Li, 2023; Ye et al., 2024). This aligns with the dynamic constructivist theory of personality, which emphasizes trait-contingent information processing styles. (3) Autonomy Support Sensitivity. The intelligent affordances of AI systems resonate strongly with high-openness employees’ autonomy needs, amplifying their perceived control over innovation processes. L. Zhang and Xu’s (2025) longitudinal study found that openness intensifies the relationship between AI autonomy support and creative self-efficacy.

Contrastingly, low-openness individuals exhibit technological myopia—they predominantly utilize AI for instrumental efficiency gains while resisting its transformative potential. This utilitarian orientation engenders either overdependence or counterproductive resistance, both undermining self-efficacy development. Accordingly, this paper proposes the following hypothesis:

**H4.**  *Openness plays a moderating role in AI usage and self-efficacy. The higher the level of openness is, the stronger the positive effect of AI usage on self-efficacy is.*

### 2.5. The Moderating Role of Job Complexity

Job complexity, conceptualized as a composite measure of cognitive demands, task diversity, and uncertainty inherent in work activities, manifests through multistep task sequences, problem space ambiguity, information processing difficulty, and decision-making autonomy (Venkataramani & Tang, 2024; Verma & Singh, 2022). High-complexity roles necessitate advanced cognitive integration capabilities, adaptive problem-solving skills, and the innovative agility to navigate ill-structured challenges (X. Qiu, 2024; Melián-González, 2024). Emerging evidence positions job complexity as a pivotal boundary condition in technology-adoption psychology (Zahmat Doost & Zhang, 2024), particularly in AI implementation contexts (Huo et al., 2025).

Empirical investigations revealed that job complexity amplifies AI’s innovation-enabling potential through dual mechanisms: (1) Cognitive Challenge Augmentation: complex tasks transform AI tools from efficiency drivers to cognitive collaborators, with employees leveraging AI’s analytical capabilities (e.g., pattern recognition, predictive modeling) to tackle novel problems, thereby reinforcing innovation self-efficacy (Dong et al., 2024; Peng & Bhaskar, 2023; Verma & Singh, 2022). (2) Resource-Demand Optimization: AI’s capacity for real-time data synthesis and multidimensional information integration creates a task–technology fit in complex environments, reducing cognitive overload while enhancing perceived competence (Huo et al., 2025).

High-complexity tasks trigger challenge appraisals rather than threat responses, framing AI integration as opportunities for skill mastery rather than job displacement risks. AI’s automation of routine subtasks liberates cognitive resources for exploratory innovation, directly enhancing self-efficacy. This aligns with Fried and Ferris’s (1987) Job Characteristics Model, which posits that complex tasks intrinsically motivate through experienced meaningfulness and knowledge of results.

Complex work demands heterogeneous information synthesis—a capability gap bridged by AI’s advanced analytics and decision-support systems. This resource-demand fit fosters positive ability attributions (Y. Li et al., 2024), particularly when AI systems demonstrate contextual adaptability. Neuroimaging evidence indicates that such synergies activate prefrontal cortex regions associated with creative confidence (N. Jia et al., 2024).

AI’s multimodal interaction capabilities and proactive task suggestions in complex workflows enhance perceived job control, a critical CET mediator between technology use and self-efficacy. Manufacturing case studies demonstrated that engineers using AI for smart factory monitoring reported 27% higher innovation agency beliefs compared to low-complexity counterparts (Dong et al., 2024).

Contrastingly, low-complexity roles engender AI-driven skill atrophy, the gradual erosion of problem-solving capacities through the over-reliance on automated systems. This deskilling dynamic suppresses self-efficacy by diminishing opportunities for mastery experiences (Gist & Mitchell, 1992). Longitudinal data revealed a significant decline in self-efficacy among low-complexity workers using AI without task redesign (Oldham & Fried, 2016). Therefore, the following hypotheses are proposed in this study:

**H5.**  *Job complexity plays a moderating role in AI usage and self-efficacy. The higher the level of job complexity is, the stronger the positive effect of AI usage on self-efficacy is.*

### 2.6. The Moderated Mediating Effects of Openness and Job Complexity

Building on CET, the mediating pathway from AI usage to innovation behavior through self-efficacy is contingent upon dispositional (openness) and contextual (job complexity) boundary conditions, forming a moderated mediation mechanism.

Employees with high openness reframe AI as cognitive scaffolding rather than disruptive automation, leveraging AI outputs as springboards for exploratory knowledge synthesis. This creative appropriation of AI tools enhances self-efficacy through the mastery of hybrid intelligence systems, while AI’s customizable interfaces satisfy autonomy needs through volitional task reconfiguration (Robinson, 2020). Openness facilitates cognitive reframing flexibility, enabling employees to reinterpret AI-generated constraints as innovation catalysts (Ye et al., 2024). Neuroimaging evidence revealed that high-openness individuals exhibit increased dorsolateral prefrontal cortex activation when integrating AI suggestions with domain knowledge, a neural correlate of self-efficacy (Shao et al., 2024). Conversely, low-openness employees exhibit algorithmic dependency (uncritically adopting AI outputs as prescriptive solutions) or defensive resistance (rejecting AI augmentation), both of which erode self-efficacy by suppressing innovation behavior (Samimi Dehkordi et al., 2025). Therefore, this study proposed the hypothesis:

**H6.**  *Openness moderates the mediating role of self-efficacy in the relationship between AI usage and innovation behavior.*

High-complexity tasks create optimal AI utility conditions; employees utilize AI’s advanced analytics (e.g., real-time pattern recognition) to address ill-defined problems, achieving a resource-demand fit that elevates competence appraisals (X. Qiu, 2024; X. Zhang et al., 2024). This aligns with the job characteristics theory, where complex tasks provide task identity and feedback significance to sustain intrinsic motivation (Melián-González, 2024; Zahmat Doost & Zhang, 2024). AI’s proactive support systems in complex contexts enhance perceived control over innovation trajectories. In low-complexity settings, AI’s substitutive automation reduces opportunities for skill demonstration, triggering self-efficacy erosion through deskilling dynamics. Longitudinal data indicated a significant decline in creative self-efficacy among low-complexity workers using un-adapted AI systems (Y. Li et al., 2024). Accordingly, this study proposed the following hypothesis:

**H7.**  *Job complexity moderates the mediating role of self-efficacy in the relationship between AI usage and innovation behavior.*

To enhance the clarity and coherence of the proposed theoretical framework, Table 1 summarizes the core constructs, conceptual definitions, associated hypotheses and empirical support, and theoretical affirmations based on CET. This structured overview facilitates a comprehensive understanding of how AI usage, individual dispositions, and task characteristics jointly influence innovation behavior.

**Figure 1:**
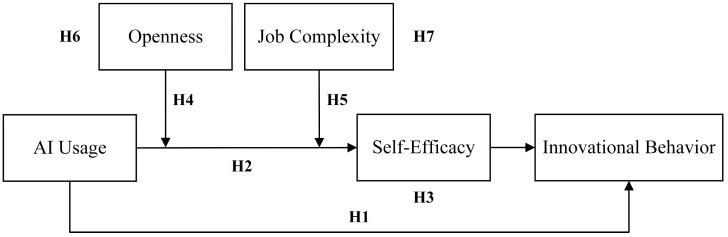
Conceptual model.

The theoretical model of this study, illustrated in Figure 1, is conceptually informed by prior research employing multi-moderator frameworks (Guo et al., 2019; F. Qiu et al., 2025). It integrates AI usage as the independent variable and innovation behavior as the dependent variable and incorporates self-efficacy as a mediator. In line with prior studies exploring conditional mechanisms, two moderators—openness and job complexity—are positioned to moderate the relationship between AI usage and self-efficacy. This modeling strategy reflects established practices in organizational behavior research involving moderated mediation structures.

## 3. Methods

### 3.1. Sample and Procedures

In this study, 13 manufacturing enterprises from four provinces, namely, Beijing, Shanghai, Hubei, and Anhui, were selected as samples, involving representative manufacturing fields such as automobile manufacturing, machinery manufacturing, electronics manufacturing, communication equipment manufacturing, and chemical manufacturing. The selected enterprises are all benchmark enterprises in their fields, which have already made abundant achievements in digital and intelligent transformation, and the application of AI technology is more in-depth.

Since this study focused on the impact of AI usage on innovation behavior, we focused on the employees of the R&D and smart manufacturing departments of these enterprises in the selection of research samples and set up a question asking whether they used AI in their work before the beginning of the study; only those who answered “yes” were allowed to enter the formal study. First, we contacted the head of the human resources department of each company to determine the scope of the survey after obtaining the consent of their supervisors. Second, online training was provided to all participants to ensure that they participated voluntarily; we promised that the responses would be anonymous and confidential and that the data would only be used for research purposes. Finally, the link to the questionnaire was distributed to the participants via WeChat groups or internal corporate email systems.

In order to minimize the effect of common method bias on the relationships between variables, the study data were collected at two different time points. At the first time point, we primarily assessed AI use, self-efficacy, job complexity, openness, and demographic variables. Respondents identified a unique four-digit code based on the last four digits of their identity ID, which was used for matching at both time points. One month after the end of Time 1, we assessed self-efficacy as well as innovative behavior at Time 2. A total of 400 questionnaires were distributed at Time 1 and 386 valid questionnaires were returned. A total of 386 questionnaires were distributed at Time 2, and 339 valid questionnaires were obtained after eliminating doubtful responses and matching the two sets of data. The overall valid response rate was 84.8%. Table 2 presents the demographic profile of the respondents.

### 3.2. Measures

All variables used a five-point Likert-type scale (1 = completely disagree to 5 = completely agree). The scale used in this article is a mature scale published in authoritative academic journals in both Chinese and English. The scales in English were adapted and adopted after being translated into Chinese by two proficient bilingual translators through translation and back-translation procedures (Brislin, 1970).

#### 3.2.1. AI Usage

Using the scale from Man Tang et al. (2022), an AI usage scale, which consisted of three items designed to evaluate AI usage was employed. Items such as “I use AI for most of my work.” and “I spend most of my time working with artificial intelligence” were included. The Cronbach’s alpha was 0.888.

#### 3.2.2. Innovation Behavior

This study utilized the items from Ng and Lucianetti’s (2016) innovation behavior scale to construct an eight-item scale for evaluating innovation behavior. Items such as “I often think about things from different perspectives” were included. The Cronbach’s alpha was 0.857.

#### 3.2.3. Self-Efficacy

We used items from Tierney and Farmer’s (2002) self-efficacy scale, which consists of seven items designed to evaluate self-efficacy, such as: “I am confident in my ability to solve problems creatively.”, “I feel that I am good at generating new and innovative ideas.”, and “I believe that I can cope with organizational change whenever it occurs.”. The Cronbach’s alpha was 0.895.

#### 3.2.4. Openness

Openness was measured using the openness subscale of Costa and McCrae’s (1992) NEO Five-Factor Inventory (NEO-FFI), which consists of 12 items (e.g., “I am curious about many things and inquisitive”), 5 of which are reverse scored (e.g., “I pay little attention to changes in my mood or feelings in different situations”). Higher scores indicate greater openness in the individual’s personality. The Cronbach’s alpha was 0.878.

#### 3.2.5. Job Complexity

Using the scale developed by Shaw and Gupta (2004), job complexity was measured with three items, such as “My job requires a lot of skills.”. The Cronbach’s alpha was 0.868.

#### 3.2.6. Controlled Variables

In line with previous relevant research (Choi et al., 2021; X. Liu & Li, 2025), this study selected common demographic variables, such as gender, age, education level, work experience, and job type, as control measures to mitigate the potential impact of demographic disparities among employees on innovation behavior.

## 4. Results

### 4.1. Common Method Biases

This study collected data at two time points and controlled for the confidentiality of the samples and the duration of responses in order to reduce the impact of common method bias. To further examine common method bias, Harman’s single-factor test was employed to conduct a factor analysis. The results revealed that the variance, which was explained by the first factor, was 25.55%, which was below the 40% threshold commonly suggested in previous research. In this study, the inclusion of unmeasured latent factors was also used to test the common method bias, and a six-factor model was constructed by adding a latent factor to the five-factor model (hypothetical model). The results showed that the model did not significantly improve (∆CFI = 0.011, ∆TLI = 0.010, ∆RESEA = 0.015). This suggests that the issue of common method bias was not severe (Ashford & Tsui, 1991).

### 4.2. Confirmatory Factor Analysis

In this study, Mplus 8.3 was used for confirmatory factor analysis, with the results shown in Table 3. In comparison with other models, the five-factor model showed a good fit (χ^2^ = 315.39, df = 160, χ^2^/df = 1.971, CFI = 0.939, TLI = 0.928, RESEA = 0.069). The results indicated that the scales demonstrated acceptable internal validity; thereby all the scales were suitable for hypothesis testing with good discriminative validity.

### 4.3. Descriptive Analysis

We used SPSS 20.0 to perform the descriptive and correlation analyses. As shown in Table 4, the AI usage was positively correlated with innovation behavior (r = 0.286, *p* < 0.01) and self-efficacy (r = 0.235, *p* < 0.01). Self-efficacy was positively correlated with innovation behavior (r = 0.403, *p* < 0.01). The correlation results provided preliminary evidence for hypothesis testing.

### 4.4. Hypotheses Tests

#### 4.4.1. Main Effect Test

The hierarchical regression model was constructed by SPSS 20.0. Table 5 shows that AI usage had a significant positive effect on innovation behavior (β = 0.282, *p* < 0.01). Thus, H1 was supported.

#### 4.4.2. Mediating Effect Test

Table 5 shows that AI usage had a significant positive effect on self-efficacy (β = 0.231, *p* < 0.01). Thus, H2 was supported. In addition, the positive effect of AI usage on innovation behavior was weakened by the addition of self-efficacy, which initially indicated that self-efficacy played a mediated role between AI usage and innovation behavior.

According to Hayes’s (2015) mediation method, the Process 3.3 plug-in of SPSS was used to conduct Bootstrap analysis to further verify the mediating effect. As shown in Table 6, the total effect of the AI usage on innovation behavior was significant because the 95% confidence interval excluded 0. The direct effect of AI usage on innovation behavior was significant because the 95% confidence interval excluded 0. The AI usage had a significant positive effect on innovation behavior through self-efficacy, and its 95% confidence interval did not contain 0, suggesting that self-efficacy played a partial mediating role. Thus, H3 was further verified.

#### 4.4.3. Moderating Effect Test

This study used the SPSS 20.0 to construct the hierarchical regression analysis to empirically test its moderating effect. Results are presented in Table 7. The results of Model 2 and Model 3 showed that the interaction of AI usage and openness had a significant positive effect on self-efficacy (β = 0.381, *p* < 0.01), indicating that openness can positively moderate the effect of AI usage on self-efficacy. Therefore, H4 was supported. The results of Model 4 and Model 5 showed that the interaction of AI usage and job complexity had a significant positive effect on self-efficacy (β = 0.334, *p* < 0.01), indicating that job complexity can positively moderate the effect of AI usage on self-efficacy. Therefore, H5 was supported.

**Figure 2:**
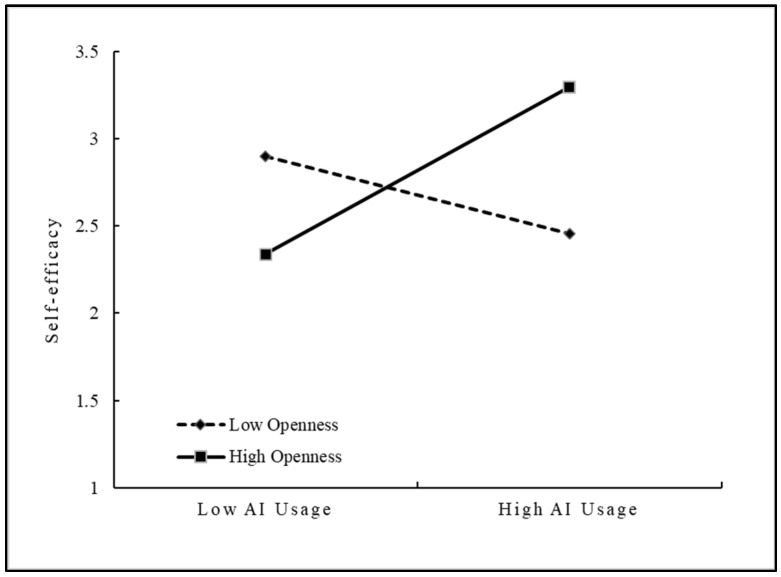
Moderating role of openness.

**Figure 3:**
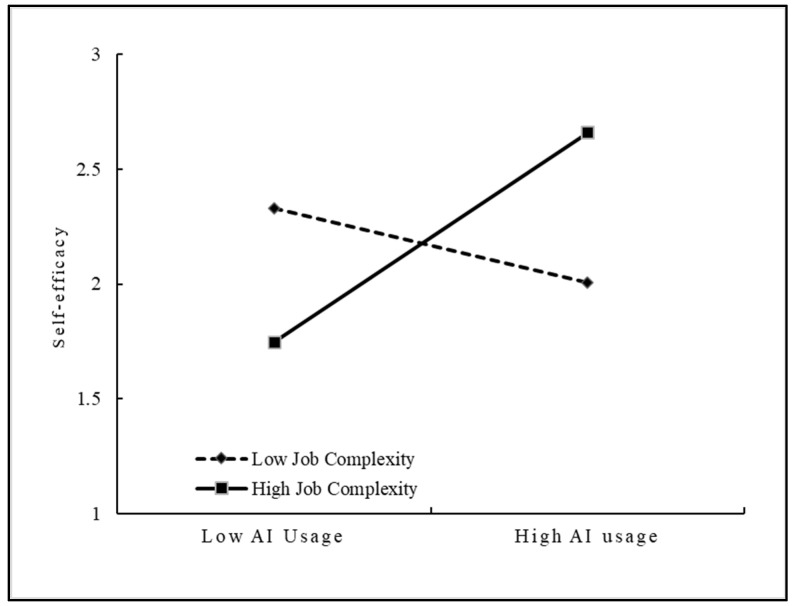
Moderating role of job complexity.

To further illustrate the moderating effects of openness and job complexity on the relationship between AI usage and self-efficacy, this study used the simple slope analysis method to draw the moderating effect diagrams with above and below the mean by one standard deviation of openness and job complexity, respectively. As shown in Figure 2, the positive effect of AI usage on self-efficacy was stronger when employees had higher openness. Figure 3 shows that the positive effect of AI usage on self-efficacy was stronger when job complexity was higher. As a result, H4 and H5 were further validated.

#### 4.4.4. Moderated Mediating Effect Test

Bootstrap analysis was conducted in the Process 3.3 plug-in to further test the moderated mediating effect. The results are presented in Table 8. It shows that if the openness was low, then the effect was −0.073 (95% bias-corrected CI = [−0.147, −0.01]), which did not include 0, and if openness was high, then the effect was 0.157 (95% bias-corrected CI = [0.085, 0.240]), which did not include 0. However, the effect of the differences between the high and low levels was 0.230 (95% bias-corrected CI = [0.125, 0.353]), which did not include 0. The moderated mediating effect of openness was significant. Therefore, H6 was supported. Similarly, if the job complexity was low, then the effect was −0.05 (95% bias-corrected CI = [−0.124, 0.012]), which included 0; if the job complexity was high, then the effect was 0.149 (95% bias corrected CI = [0.076, 0.230]), which did not include 0; finally, the effect of the differences between the high and low levels was 0.202 (95% bias-corrected CI = [0.0983, 0.3188]). This proves that the moderated mediating effect of the job complexity was significant. Therefore, H7 was supported.

## 5. Discussion

Based on the theory of cognitive evaluation, this study attempted to bridge the research gap by revealing the underlying mechanism between AI usage and innovation behavior while also establishing a comprehensive theoretical model to interpret the process in a context that considers both employee personality traits and job characteristics. The following conclusions were drawn.

Firstly, this study found the facilitating effect of AI usage on employees’ innovative behavior, while revealing the mediating role of self-efficacy in this process. Specifically, AI usage enhances employees’ confidence in their own innovativeness by boosting their sense of competence (e.g., data-driven decision support) and autonomy (e.g., flexible task control), which in turn drives innovative behavior. This finding is consistent with the core idea of cognitive evaluation theory that external technological interventions activate intrinsic motivation by satisfying individuals’ needs for autonomy and sense of competence (Tierney & Farmer, 2011). However, unlike the “AI substitution threatens to inhibit innovation” view proposed by some studies (Liang et al., 2022), this study found that, when AI usage is positively matched to employees’ cognitive appraisal processes (e.g., empowerment rather than substitution), its negative impacts may be attenuated or even reversed. For example, AI’s real-time feedback and task reframing features (Ogink & Dong, 2019) can counteract the negative effects of blunted skills or job insecurity by enhancing self-efficacy. This finding provides an integrative perspective to the current contradictory conclusions about AI and innovation behavior: the impact of AI is not unidirectional but depends on how it reconfigures employees’ perceptions of the value of technology through psychological mechanisms (e.g., self-efficacy).

Secondly, openness plays a significant positive moderating role between AI usage and self-efficacy. Employees with high openness are more likely to view AI as a cognitive extension tool (M. Li & Bitterly, 2024) and to strengthen their confidence in their ability to innovate by proactively integrating AI outputs with their own knowledge for secondary innovation (Leiblein et al., 2023). This moderating effect fits the individual differences perspective of CET: the level of openness influences the individual’s challenge-threat assessment framework of AI usage. High-openness individuals focus more on the enabling potential of AI than on substitution risks, thereby activating intrinsic motivation. In contrast, low-openness employees may perceive AI as a threat due to cognitive rigidity, resulting in limited self-efficacy enhancement (L. Zhang & Xu, 2025). This finding echoes Z. Li et al.’s (2024) study. High-openness individuals’ ability to “cognitively reconfigure” enables them to flexibly utilize heterogeneous information (e.g., data analytics) from AI to think outside the box, whereas low-openness individuals may be inhibited from creative exploration due to their dependence on AI output.

Thirdly, job complexity plays a positive moderating role between AI usage and self-efficacy. When job complexity is high, AI significantly increases employees’ self-efficacy by automating repetitive processes and providing decision support, freeing up their cognitive resources to focus on creative problem-solving (N. Jia et al., 2024). Conversely, the substitution effect of AI usage in low-complexity tasks may trigger “skill blunting”, reducing employees’ self-efficacy. This finding is consistent with the job characteristics model’s prediction that high job complexity enhances employees’ perceptions of AI-enabled value by granting them decision-making freedom and task integrity (N. Jia et al., 2024). Meanwhile, AI is not only an instrumental support but also a cognitive collaboration partner in complex tasks. AI’s multimodal interaction capabilities can help employees quickly integrate information in complex systems and provide feedback in real time, a process that strengthens their sense of “resource-need” matching and further improves their innovation behavior through self-efficacy (X. Zhang et al., 2024).

### 5.1. Theoretical Implications

First, we revealed the mediating role of self-efficacy between AI usage and innovative behavior and deepened the influence mechanism of AI usage. It revealed how AI usage activates self-efficacy through the paths of competence and autonomy, which in turn improve innovative behavior, forming a complete chain of “technical characteristics—psychological motivation—behavioral outcomes”. Specifically, the intelligence and adaptability of AI can satisfy employees’ needs for autonomy and sense of competence, strengthen their belief in their own innovative capabilities, and then promote innovative behavior. This finding deepens research on the psychological transmission mechanisms of technology adoption. In addition, the study extends Gist and Mitchell’s theory of self-efficacy to AI scenarios, reinforcing the value of self-efficacy in the age of AI and providing a direction for the future exploration of psychological mechanisms in human–AI symbiosis.

Second, we expanded the application of cognitive evaluation theory by reconstructing the technology effect framework from the perspective of individual differences and contextual interaction. By introducing openness and job complexity as moderating variables, this study expands the explanatory boundaries of cognitive evaluation theory and constructs a dynamic contextualized theoretical framework. Traditional CET emphasizes that the external environment influences behavioral motivation by satisfying basic psychological needs but does not adequately explain how individual differences and task characteristics moderate this process. The cognitive reframing effect of openness and the task–technology matching effect of job complexity play a key role in explaining the process by which technology stimulates individuals’ cognition and motivation and thus influences behavioral outcomes, enriching the individual difference perspective of CET while emphasizing the interactive logic of synergy between task characteristics and AI.

Third, aiming at the contradictory conclusions of the current relationship between AI and innovation behavior, this study provides an integrated perspective for the controversy through the refinement of boundary conditions. We proposed an “openness–complexity” dual-regulation model, which points out that the direction of AI’s effects on innovation depends on the interaction between individual traits and task characteristics. By revealing the context-dependent nature of psychological mechanisms (e.g., self-efficacy), we suggest that the negative effects of AI can be attenuated or even reversed by optimizing individual–task–technology matching. This framework not only reconciles the opposing views of “empowerment” and “substitution” but also provides a theoretical basis for contingency strategies for AI technology management.

### 5.2. Practical Implications

First, establish an AI talent management strategy based on personality traits. Given the significant moderating effect of openness on AI usage, companies should prioritize the selection or cultivation of high-openness employees to participate in AI-driven innovation projects. For employees, organizations should encourage those with low openness to come into contact with AI application scenarios and gradually improve their technical adaptability and cognitive reconstruction ability. For managers, personality assessment tools can be introduced during recruitment to screen candidates who are inclusive of new things and have high cognitive flexibility. At the organizational level, companies may establish a “human–AI collaboration mentorship system”, where high-openness employees lead the team to explore the creative usage of AI, thereby stimulating the sense of innovation effectiveness of the whole staff.

Second, achieve synergistic optimization of task design and AI deployment. Based on the moderating effect of job complexity, enterprises need to maximize the enabling value of AI through task redesign. For employees, those in high-complexity jobs should be supported with enhanced AI decision support functions (e.g., dynamic data visualization, multimodal interactive interfaces) to help them efficiently integrate information and release cognitive resources. Those in low-complexity positions can benefit from “AI + Human” hybrid task models (e.g., AI handles standardized processes, and employees are responsible for anomaly detection and optimization) to maintain task engagement and a sense of value. For managers, task autonomy should be increased to enhance employees’ sense of control and interactivity with AI tools, and continuous feedback loops should be established to adjust AI deployment. Organizationally, companies should implement differentiated task structures tailored to job complexity and ensure that AI tools are accessible, adaptable, and properly aligned with employee roles.

Third, build a supportive organizational environment and training system. In order to strengthen the positive impact of AI usage on self-efficacy, employees should be provided with customized training programs and AI application courses based on their job complexity and technical readiness. Managers should actively gather feedback on employees’ AI experiences and lead cultural efforts that reduce substitution anxiety and encourage experimentation. At the organizational level, it is advisable to establish an “AI innovation lab” to facilitate cross-departmental collaboration in AI solution development and disseminate successful use cases through internal knowledge-sharing sessions. In addition, regular reviews of technology deployment strategies can help ensure continuous alignment with employees’ evolving needs.

### 5.3. Limitations and Future Directions

This study has several limitations that set the stage for promising future studies. First, this study took manufacturing employees as the research object, and the generalizability of the conclusions in technology-intensive industries needs to be further verified. For example, the application scenarios of AI in the service industry may rely more on emotional intelligence rather than analysis capabilities and there may be differences in the mechanisms influencing their innovative behavior. Cross-industry comparative studies can be conducted in the future to explore the differences that exist in the effects of AI usage by different industry characteristics or enterprise size.

Second, despite the use of methods such as two-stage data collection to control for common method bias, variable measurement still relies on self-assessment scales. Multi-source validation could be introduced in the future with other-rated data or objective fingers. A longitudinal design could reveal the long-term effects of AI usage. In addition, other research methods such as case studies or experimental studies could be further employed.

Finally, this study focused on the mediating role of self-efficacy and the moderating effect of openness and job complexity, but other potential mechanisms remain to be explored. In the future, multidimensional mediation models can be constructed from different theoretical perspectives. In addition, future research can expand the research scenario to human–AI collaborative scenarios, focus on the topics of human–AI interaction sense of efficacy and human–AI collaborative innovation, and promote the in-depth application of AI in the field of enterprise and employee innovation.

## Figures and Tables

**Table 1:** Affirmation of key variables and hypotheses.

Variables	Conceptions	Hypotheses and Empirical Support	Theoretical Support
Innovation behavior	It refers to employees’ proactive efforts to identify improvement opportunities and implement novel solutions.	H1 (Zang et al., 2024; Y. Liu et al., 2024; Zheng et al., 2025)	Based on cognitive evaluation theory (CET), AI usage enhances employees’ perceived competence by providing task support and informational feedback. This increased self-efficacy, in turn, promotes innovation behavior. Individual traits (openness) and task characteristics (job complexity) moderate how AI influences self-efficacy and the strength of the indirect effect on innovation. CET provides a coherent framework linking AI usage, self-efficacy, and innovation behavior
AI usage	The extent to which employees leverage AI technologies to achieve work objectives.	H2 (Chen et al., 2025; Eissa & Lester, 2025; G. Jia et al., 2025)	
Self-efficacy	An individual’s belief in their ability to plan and perform actions to achieve specific goals.	H3 (Yuan et al., 2025; K. L. Huang et al., 2024; D. Wang et al., 2025)	
Openness	Reflects individual differences in creativity, curiosity, and preference for novelty and aesthetics.	H4 (Pillai et al., 2024; X. Zhang et al., 2024; L. Zhang & Xu, 2025) H6 (Samimi Dehkordi et al., 2025; Ye et al., 2024; Shao et al., 2024)	
Job complexity	A composite of cognitive load, task variety, and uncertainty, reflected in multistep processes, ambiguous problems, complex information, and autonomous decision-making.	H5 (X. Qiu, 2024; Huo et al., 2025; Y. Li et al., 2024) H7 (Melián-González, 2024; Zahmat Doost & Zhang, 2024; Dong et al., 2024)	

**Table 2:** Demographic characteristics of the respondents.

Variable	Category	Frequency	Percentage (%)	Cumulative (%)
Gender	Male	224	66.08	66.08
	Female	115	33.92	100.00
Age	≤25 years	50	14.75	14.75
	26–30 years	90	26.55	41.30
	31–40 years	110	32.45	73.75
	41–50 years	60	17.70	91.45
	≥51 years	29	8.55	100.00
Education level	High school or below	20	5.90	5.90
	Associate’s degree	91	26.84	32.74
	Bachelor’s degree	160	47.20	79.94
	Master’s degree	54	15.93	95.87
	Doctorate or above	14	4.13	100.00
Work experience	1 year or less	35	10.32	10.32
	2–5 years	204	60.18	70.50
	6–10 years	52	15.34	85.84
	11–15 years	24	7.08	92.92
	More than 15 years	24	7.08	100.00
Job type	Frontline staff	90	26.55	26.55
	General employees	80	23.60	50.15
	Middle-level managers	70	20.65	70.80
	Senior managers	50	14.75	85.55
	Technical specialists	49	14.45	100.00

**Table 3:** Confirmatory factor analysis of model fit.

Model	Factors	χ^2^	df	χ^2^/df	RESEA	CFI	TLI
Five-factor model	AU, SE, OP, JC, IB	315.392	160	1.971	0.067	0.939	0.928
Four-factor model	AU, SE, OP + JC, IB	715.312	164	4.362	0.125	0.784	0.758
Three-factor model	AU, SE + OP + JC, IB	1215.453	167	7.278	0.175	0.589	0.533
Two-factor model	AU + SE + OP + JC, IB	1683.633	169	9.962	0.204	0.407	0.333
One-factor model	AU + SE + OP + JC + IB	1992.859	170	11.723	0.223	0.286	0.202

Notes: N = 339; AU = AI usage; SE = self-efficacy; OP = openness; JC = job complexity; and IB = innovation behavior. The following is consistent; + means merged into one factor.

**Table 4:** Descriptive analysis of all variables.

Variable	1	2	3	4	5	6	7	8	9	10
1 Gender										
2 Age	−0.049									
3 Education level	−0.017	−0.145 *								
4 Work experience	−0.025	0.506 **	−0.010							
5 Job type	−0.090	−0.043	−0.031	−0.137 *						
6 AU	−0.055	−0.008	−0.009	0.027	−0.020					
7 IB	−0.053	−0.043	−0.073	−0.023	−0.028	0.286 **				
8 SE	−0.076	0.067	−0.012	0.032	−0.058	0.235 **	0.403 **			
9 OP	−0.069	−0.047	0.038	0.009	−0.020	0.060	0.064	0.131		
10 JC	0.092	−0.138 *	0.054	−0.086	0.004	0.042	0.075	0.072	0.252 **	
Mean	1.660	4.620	2.360	3.390	4.590	2.881	2.828	2.821	2.947	3.028
SD	0.476	2.348	0.714	1.811	2.592	1.058	0.974	1.074	1.014	1.008

Notes: * *p* < 0.05; ** *p* < 0.01; N = 339.

**Table 5:** The results of hierarchical regression model.

Variable	Self-Efficacy		Innovation Behavior		
	Model 1	Model 2	Model 3	Model 4	Model 5
Gender	−0.080	−0.067	−0.610	−0.045	−0.210
Age	0.066	0.074	−0.058	−0.049	−0.075
Education level	−0.006	−0.003	−0.084	−0.080	−0.079
Work experience	−0.014	−0.023	−0.003	−0.014	−0.005
Job type	−0.065	−0.060	−0.040	−0.033	−0.012
AU		0.231 **		0.282 **	0.200 **
SE					0.358 **
F	0.595	11.937 **	0.562	18.359 **	31.703 **
R2	0.014	0.067	0.013	0.092	0.212
∆R2	0.014	0.053	0.013	0.079	0.120

Notes: ** *p* < 0.01; N = 339.

**Table 6:** Bootstrapping mediation testing results.

Pathway	Effect	SE	95% CI	
			Low	High
Total effect	0.282	0.039	0.206	0.388
Direct effect	0.200	0.027	0.163	0.268
AU→SE→IB	0.082	0.023	0.022	0.161

**Table 7:** Results of the moderation effects.

Variable	Self-Efficacy				
	Model 1	Model 2	Model 3	Model 4	Model 5
Gender	−0.080	−0.059	−0.021	−0.074	−0.046
Age	0.066	0.073	0.111	0.083	0.096
Education level	−0.006	−0.007	0.018	−0.006	0.043
Work experience	−0.014	−0.023	−0.056	−0.021	−0.024
Job type	−0.065	−0.057	−0.039	−0.060	−0.028
AU		0.224 **	0.127 *	0.227 **	0.144 *
OP		0.113	0.067		
AU × OP			0.381 **		
JC				0.080	0.016
AU × JC					0.334 **
F	0.595	7.464 **	33.894 **	6.676 **	24.193 **
R2	0.014	0.080	0.209	0.073	0.170
∆R2	0.014	0.066	0.129	0.059	0.097

Notes: * *p* < 0.05; ** *p* < 0.01; N = 339.

**Table 8:** Moderated mediation effect of openness and job complexity.

Path	Mediator			Moderated Mediation	
	Moderator	Effect	95% CI	Index	(CI)
AU-SE-IB	Low OP (−1 SD)	−0.0732	[−0.1474, −0.0104]		
	High OP (+1 SD)	0.1570	[0.0852, 0.2398]	0.1135	[0.0614, 0.1738]
	Difference group	0.2302	[0.1245, 0.3526]		
	Low JC (−1 SD)	−0.0534	[−0.1237, 0.0115]		
	High JC (+1 SD)	0.1486	[0.0759, 0.2297]	0.1002	[0.0488, 0.1582]
	Difference group	0.2020	[0.0983, 0.3188]		

## Data Availability

The data presented in this study are available upon request from the corresponding author.
